# Association between ovarian reserve and preeclampsia: a cohort study

**DOI:** 10.1186/s12884-019-2578-y

**Published:** 2019-11-21

**Authors:** Hadi Erfani, Maryam Rahmati, Mohammad Ali Mansournia, Fereidoun Azizi, Seyed Ali Montazeri, Alireza A. Shamshirsaz, Fahimeh Ramezani Tehrani

**Affiliations:** 1 Department of Obstetrics and Gynecology, Baylor College of Medicine, Houston, TX USA; 2 Reproductive Endocrinology Research Center, Research Institute for Endocrine Sciences, Shahid Beheshti University of Medical Sciences, 24 Parvaneh, Yaman Street, Velenjak,, P.O. Box:19395-4763, Tehran, 1985717413 Iran; 3 Department of Epidemiology and Biostatistics, School of Public Health, Tehran University of Medical Sciences, Tehran, Iran; 4 Endocrine Research Center, Research Institute for Endocrine Sciences, Shahid Beheshti University of Medical Sciences, Tehran, Iran

**Keywords:** Anti-Mullerian hormone, Ovarian reserve, Pre-Eclampsia

## Abstract

**Background:**

The risk of cardiovascular disease in women increases after menopause. It has been shown that women with lower pre-menopausal ovarian reserve may experience increased cardiovascular risk. We sought to determine whether there is any association between ovarian reserve, as assessed by Anti-Mullerian hormone (AMH), and preeclampsia (PE).

**Methods:**

Subjects of this study were selected from among participants of the Tehran Lipid and Glucose Study (TLGS), a population-based cohort with a 15-year follow-up (1998–2014). Out of 2412 women aged 20–50 years, there were 781 women who met eligibility criteria, including having comprehensive data on their reproductive assessment and ovarian reserve status, identified based on age-specific AMH levels according to the exponential–normal three-parameter model that was measured before pregnancy.

There were 80 and 701 participants in the preeclampsia and non-PE groups, respectively. The association between dichotomous outcome variable PE and age-specific AMH quartiles was evaluated using pooled logistic regression.

**Results:**

PE was observed in 23 (11.1%), 12 (6.4%), 26 (13.3%) and 19 (10%) women in the 1st, 2nd, 3rd and 4th quartiles of pre-pregnancy age-specific AMH, respectively (*P* = 0.16). Median and inter-quartile range of serum AMH levels was 1.05 (0.36–2.2) mg/L in women who experienced PE compared with 0.85 (0.28–2.1) mg/L in women with normotensive pregnancies (*P* = 0.53). Based on the pooled logistic regression analysis, the effect of age-specific AMH quartiles on PE progression (adjusted for age, BMI, smoking status, and family history of hypertension) were not significant (OR_1st vs 4th_: 1.5, *P*-value: 0.1, CI: (0.9, 2.4)).

**Conclusions:**

Age-specific AMH may not be a suitable marker for prediction of PE. Further longitudinal studies, considering pre-conception measurement of AMH, are recommended for better interpretation of the association between ovarian reserve status and PE.

## Background

Pregnancy induced hypertension/Pre-Eclampsia (PE) is a major cause of maternal and fetal morbidity and mortality. Hypertensive disorders of pregnancy occur in 3–10% of all pregnancies, with preeclampsia playing a role in approximately 10% of maternal deaths in the United States [1–3]. Thus, the identification of risk factors for hypertensive disease in pregnancy is essential, and might offer a better understanding of the underlying pathophysiology of this condition.

Anti-Mullerian Hormone (AMH) is a highly glycosylated homodimeric glycoprotein which is produced in the somatic cells of the ovaries; a member of the transforming growth factor beta family secreted by the granulosa cells of the ovarian follicles [4]. Previous studies suggest that serum AMH is a good indicator of ovarian follicular reserve and a single measurement can both reflect ovulatory decline and reliably predict the age of menopause [5–8]. In addition several studies have addressed the potential association of low ovarian reserve with cardiovascular risks [9–11]. A study by de Kat et al., in 2017 indicated that AMH trajectories in women are associated with cardiovascular risks; therefore, the decline of circulating AMH levels may be part of the pathophysiology of increased cardiovascular risk attributed to the early menopause [12]. In another study Kim et al., proposed belief that in midlife women with type 1 diabetes AMH has slight but significant association with subclinical measures of atherosclerosis [13].

These observations suggest the possibility that serum AMH levels might also be predictive of pregnancy induced hypertension/ PE, a pre-menopausal cardiovascular disease of major clinical importance, particularly since women with PE are at risk for later cardiovascular disease. Several previous studies have suggested a link between AMH levels and PE [14–16].

In this study, we sought to determine whether ovarian reserve status, as indicated by serum concentrations of AMH or age specific AMH predicts PE using data from a 15-year population-based cohort.

## Methods

### Study subjects

The ethics committee of the Research Institute for Endocrine Sciences approved the study (IR.SBMU.ENDOCRINE.REC.1398.009) and written informed consent was obtained from all subjects before initiation of the study. Data was obtained from the Tehran Lipid and Glucose Study (TLGS), an ongoing prospective study in Tehran, Iran, initiated in 1998 [17]. To date TLGS has completed five phases at 3-year intervals (phase 1: 1999–2001, phase 2: 2002–2005, phase 3: 2005–2008, phase 4: 2008–2011 and phase 5: 2011–2014). Current data are available for five phases, including baseline and four follow-ups. TLGS involves 15,005 subjects, aged ≥3 years, who were selected from a geographically defined population using multi-stage cluster sampling. At the time of data collection (both base line and follow-ups) women were interviewed by trained personnel using pretested questionnaires including information on demographic and lifestyle variables, smoking habits, various risk factors for non-communicable diseases, family history of hypertension and medical and reproductive histories; all clinical, and anthropometric parameters included weight and Waist Circumference (WC) were measured by interviewers, as well. WC was measured with an unstretched tape measure at the level of the umbilicus, without any pressure to the body surface. Systolic Blood Pressure (SBP) and Diastolic Blood Pressure (DBP) were measured twice in a seated position after a 15-min rest period. At each visit, women were asked about having any experience of PE, using a validated self-reporting questionnaire [18, 19]; when the women were not sure of the diagnosis their medical documents were referred to.

A blood sample was taken from all participants between 7:00 am and 9:00 am after a 12-h overnight fast. Biochemical assessments were performed at the TLGS research laboratory on the day of blood collection. Back up samples were stored at − 80 degree of centigrade.

Triglyceride (TG) levels were assayed using glycerol phosphate. Total Cholesterol (TC) was assayed using the enzymatic colorimetric method with cholesterol esterase and cholesterol oxidase. The level of High-density Lipoprotein Cholesterol (HDL-C) was measured after precipitation of the apolipoprotein B (apo B)-containing lipoproteins with phosphotungstic acid. We used a modified Friedewald equation to calculate Low-density Lipoprotein Cholesterol (LDL-C). Fasting plasma glucose (FPG) and 2-h post-challenge plasma glucose (2 h-PCPG) were measured using an enzymatic colorimetric method with glucose oxidase; inter- and intra-assay Coefficients of Variations (CVs) at baseline and follow-up phases were both < 2.3%. All metabolic analyses were performed using related kits and a Selecta 2 autoanalyzer. Intra-assay and inter-assay CVs for TG, TC, HDL-C, and LDL-C were less than 2.1, 1.9, 3, and 3%, respectively.

Serum concentration of AMH was measured in stored samples at the time of recruitment by the two-site enzyme immunoassay (EIA) method using Gen II kit (intra- and inter-assay CVs were 1.9 and 2.0%, respectively) and sunrise ELISA reader. More details on measurements have been previously published elsewhere [10].

**Fig. 1:**
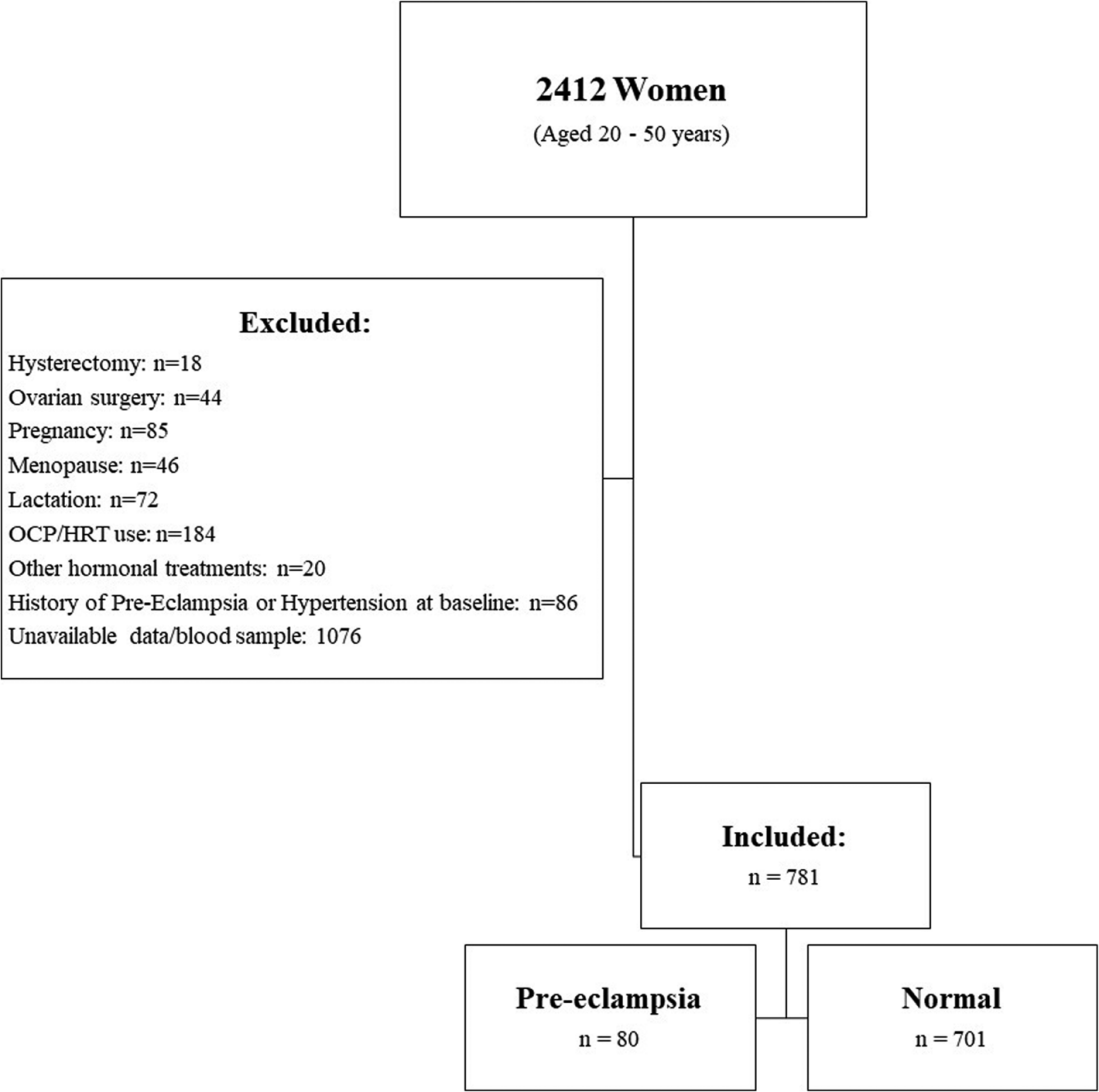
Summary of case recruitment for the study

Our study population included all women who met the following eligibility criteria: [1] age between 20 and 50 years, [2] having regular cycles at the time of enrollment, [3] having history of normal fertility and delivery (at least one term pregnancy within 1 year of stopping contraception) without having a history of pregnancy after Assisted Reproductive Technologies (ART), and [5] having no history of endocrine problems, hysterectomy, oophorectomy, or any other surgeries on ovaries. There were 1015 subjects who met the inclusion criteria; after exclusion of those with uncertain data regarding history of PE, those with history of PE or chronic hypertension at initiation of the study (*n* = 112), data of 781 women remained for inclusion in the present study (Fig. 1).

### Definitions

The normal-based methodology used to calculate age-specific AMH percentiles has been described previously in detail by Altman and Chitty [20] and Royston and Wright [21, 22]. Age-specific AMH was estimated using the exponential–normal three-parameter model. Our previous study depicts cut-off values for women of each specific age for defining the age-specific AMH quartiles [23]. PE was defined based on the international standard criteria [24]. In our country, PE diagnosis is as a part of routine prenatal care that is determined based on the standard definition of onset of a BP level ≥ 140/ 90 mmHg with proteinuria > 0.3 g/24 h after 20 weeks’ gestation. At the time of data collection, women were asked about their history of PE based on a specific validated self-reporting questionnaire [25]. For patients who have no accurate information about their PE diagnosis, we checked their summary from hospital records.

### Statistical analysis

All continuous variables were checked for normality using the one-sample Kolmogorov–Smirnoff test, and expressed as mean ± standard deviation, or median with inter-quartile range (IQ25–75). To normalize the distribution of AMH, log transformation was used. Characteristics of women at the time of recruitment were compared between those experienced PE during follow-ups and those who did not experience (non-PE) using either two independent sample t-test or the Mann-Whitney U-test. The categorical variables, expressed as percentages, were compared using Pearson’s *χ*^2^ test.

A Receiver Operating Characteristics (ROC) curve was used for assessing predictive power of AMH for event of PE, and the Area Under Curve (AUC) was calculated. To explore the association between PE status and serum AMH levels linear regression analysis with censoring, based on the Buckley-James method (on log-transformed AMH) was used and adjusted for the confounders such as smoking status (ever/never), BMI (kg/m2), SBP (mmHg) and family history of hypertension (yes/no). If the AMH level was undetectable (<.16 mg/L), AMH values were censored [14]. To compare the potential nonlinear relationship between AMH and age/BMI between those who experienced and those who did not experience PE, fractional polynomial function was used; the relationship has been depicted graphically.

We also used pooled logistic regression to assess the association between dichotomous outcome variable PE and the time-dependent covariates as the data was interval censored and time to PE was not known, and to calculate odds ratios [26]. This model treats every interval as a mini follow up study, pools the observations of all intervals together into one pooled sample and does a logistic regression on the pooled dataset; it has been adjusted for above mentioned confounders.

We used Stata Statistical Software (Release 14. College Station, TX: Stata Corp LP) and a *P*-value of < 0.05 was considered statistically significant.

## Results

Baseline characteristics of the study population are summarized in Table 1. Mean WC, SBP, DBP, and smoking habit were different between the 2 groups (Table 1).

**Table 1:** Characteristics of Participants in women experienced preeclampsia during follow-ups with those not experienced

	PE	Non-PE	*P*-Value
	*N* = 80	*N* = 701	
Age at AMH measurement (year), mean ± SD	36.9 ± 6.7	37.6 ± 6.5	0.32
BMI (Kg/m^2^), mean ± SD	27.9 ± 4.3	27.1 ± 4.4	0.10
Waist circumference (cm), mean ± SD	89.7 ± 10.9	86.0 ± 10.2	**0.003**
WHR, median (IQr)	0.8 (0.8–0.9)	0.8 (0.8–0.9)	0.08
Parity, mean ± SD	2.5 ± 1.2	2.6 ± 1.3	0.44
Number of abortions, mean ± SD	0.5 ± 0.9	0.4 ± 0.7	0.35
Higher education, n (%)	43 (53.8%)	356 (50.8%)	0.61
Ever Smoker, n (%)	9 (11.3%)	31 (4.4%)	**0.01**
SBP(mmHg), mean ± SD	114.0 ± 11.8	109.1 ± 10.4	**0.001**
DBP(mmHg), mean ± SD	76.9 ± 7.4	73.6 ± 7.6	**< 0.001**
Cholesterol (mg/L), mean ± SD	198.8 ± 37.1	195.4 ± 3.7	0.40
Tg (mg/dL), median (IQr)	132 (93–194)	114 (83.5–162)	0.06
LDL (mg/L), mean ± SD	127.9 ± 31.2	125.2 ± 30.1	0.46
HDL (mg/L), mean ± SD	42.5 ± 8.5	44.0 ± 10.1	0.19
Fasting BG (g/dl), mean ± SD	96.9 ± 41.0	90.3 ± 20.5	0.16
AMH level (mg/L), median (IQr)	1.05 (0.36–2.2)	0.85 (0.28–2.1)	0.53
Non-detectable AMH levels, n (%)	13 (16.3)	114 (16.3)	0.90

*PE* experienced preeclampsia during follow ups; *non-PE* not experienced preeclampsia during follow ups; *AMH* anti-mullerian hormone; *BMI* body mass index; *WHR* waist to hip ratio; *SBP* systolic blood pressure; *DBP* diastolic blood pressure; *Tg* triglyceride; *LDL* low density lipoprotein; *HDH* high density lipoprotein; *BG* blood glucose

Higher education was defined as equivalent of the completion of K-12 or above

Variables are reported as mean ± SD (Student t test), median(IQr) (Mann-Whitney U test) or n(%) Chi square test as appropriate

Bold values are considered significant

During follow-ups PE occurred in 23 (11.1%), 12 (6.4%), 26 (13.3%) and 19 (10%) women in the 1st, 2nd, 3rd and 4th quartiles of age-specific AMH, respectively (*P* = 0.16).

The median and inter-quartile range of serum AMH level was 1.05 (0.36–2.2) mg/L in women who experience PE, compared with 0.85 (0.28–2.1) mg/L in women with normotensive pregnancies (*P* = 0.53).

On the basis of the Buckley-James regression method, the unadjusted relative difference in AMH levels comparing PE with non-PE groups was 0.06 with 95% CI (− 0.26, 0.14); in other words, we determined that the baseline AMH levels of women who experienced PE did not differ significantly from those of women who did not experienced PE women with experienced PE, even after adjustments for age, BMI, smoking status, and family history of hypertension (Table 2).

**Table 2:** Association between preeclampsia status and baseline serum concentration of AMH levels using the Buckley-James method^a^ (*n* = 781)

	Relative difference in AMH, (mg/L) (95% CI)
PE Vs. non-PE	0.06 (−0.26, 0.14)
Adjusted for age(year)	0.01 (−0.26, 0.24)
Adjusted for age(year) and BMI(kg/m^2^)	0.0 (−0.25, 0.25)
Adjusted for age(year), BMI(kg/m^2^) and smoking status(ever/never)	0.01 (−0.26, 0.24)
Adjusted for age(year), BMI(kg/m2), smoking status (ever/never)and family history of hypertension(yes/no)	0.02 (−0.23, 0.27)

*PE* experienced preeclampsia during follow ups; *non-PE* not experienced preeclampsia during follow ups; *BMI* body mass index; *AMH* anti-mullerian hormone

^a^ Buckley-James method was used as linear regression analysis with censoring on log-transformed AMH,if the AMH level was undetectable (<.16 mg/L), AMH values were censored

**Fig. 2:**
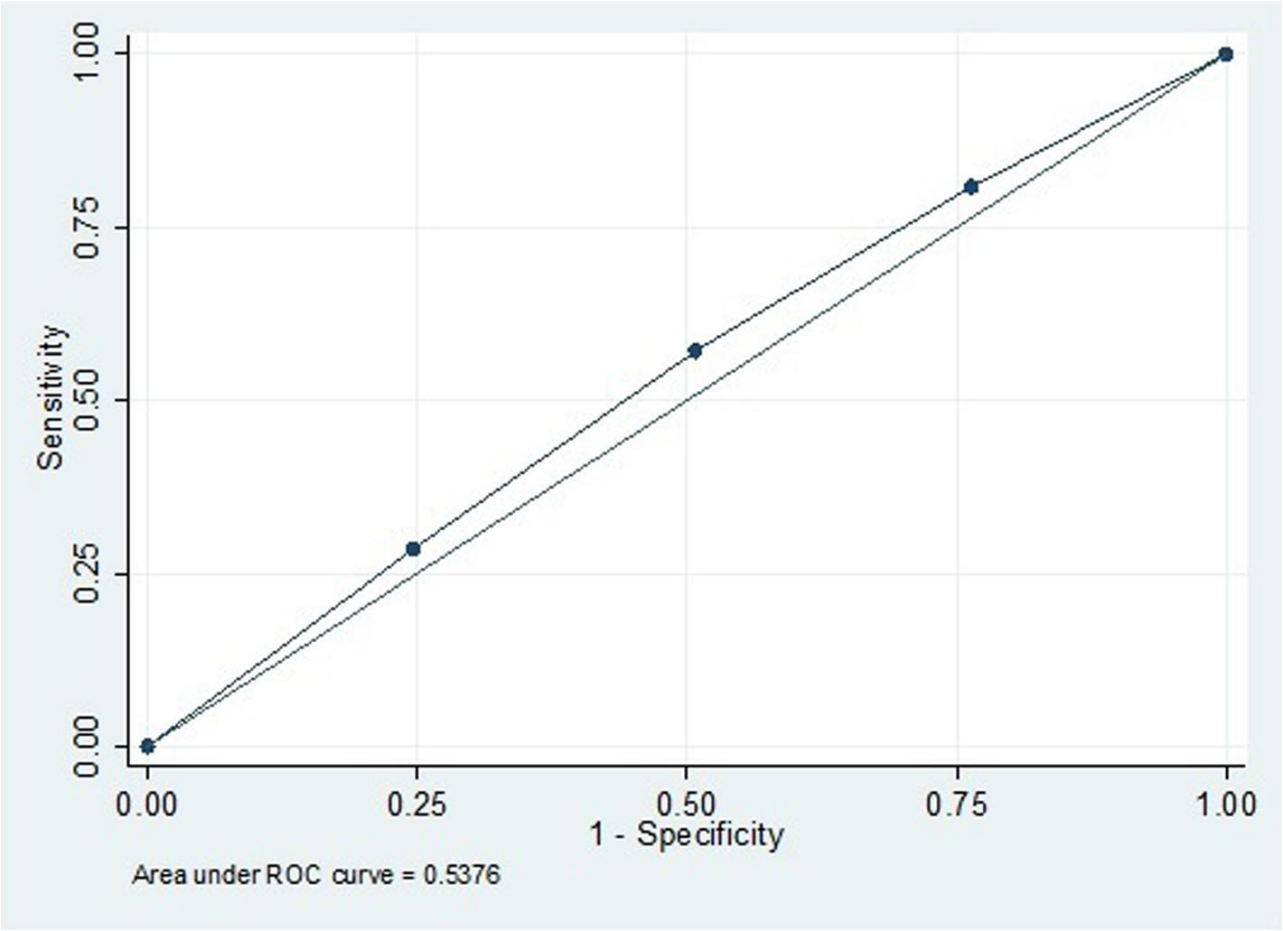
ROC curve for age-specific AMH as a predictor of preeclampsia

**Fig. 3:**
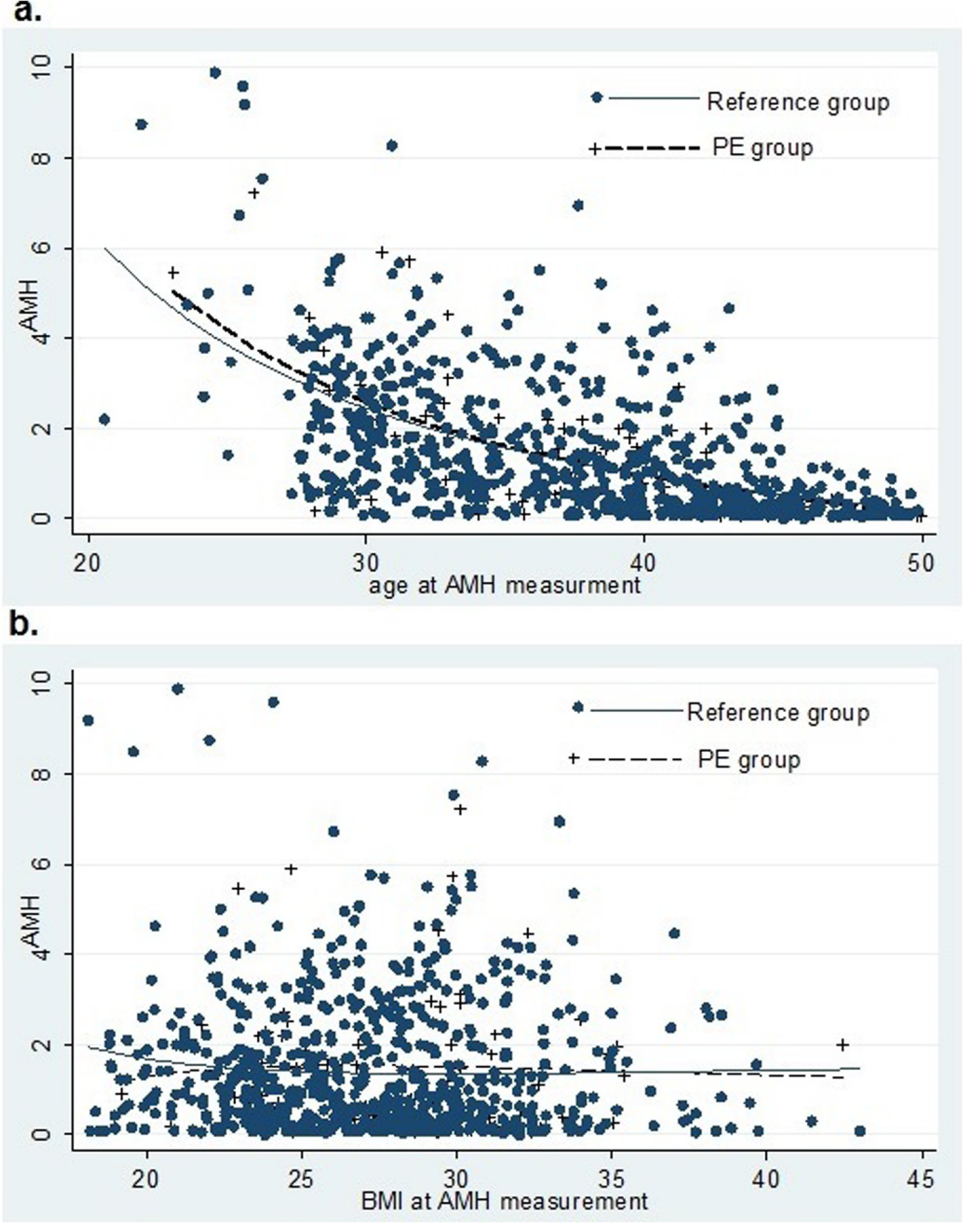
Age related (**a**) and BMI related (**b**) AMH in women with PE compared with that of a reference group. The serum AMH level is plotted on a logarithmic scale

According to the pooled logistic regression analysis, the effects of age-specific AMH quartiles on PE progression (adjusted for age, BMI, smoking status, and family history of hypertension) were not significant (OR_1st vs 4th_: 1.5, *P*-value: 0.1, CI: (0.9, 2.4)) (Table 3). Neither did ROC analysis of age-specific AMH show any predictive performance for PE; AUC (95% CI) was 0.54 (0.45–0.63) (*P* = 0.40) (Fig. 2). Decrease in AMH levels, with increasing age and BMI, was not statistically different between the two groups (PE and non-PE) (Figs. 3.a and 3.b).

**Table 3:** Adjusted pooled logistic regression analysis for progression of preeclampsia according to age-specific AMH quartiles

	aOR (95% CI)	*p*-value
^a^Age-specific AMH		
1st Quartile	1.48 (0.89, 2.48)	0.1
2nd Quartile	1.02 (0.74–2.54)	0.8
3rd Quartile	0.97 (0.54–1.74)	0.9
4th Quartile	1(reference group)	

*aOR* adjusted odds ratio; *AMH* anti-mullerian hormone; *BMI* body mass index

^a^Age-specific AMH was calculated using the exponential–normal three-parameter model

Model was adjusted for smoking status, BMI, systolic blood pressure and family history of hypertension

## Discussion

In this study we found that ovarian reserve status, estimated using AMH serum concentrations may not be useful in the prediction of increased risk for subsequent PE.

Previous animal studies have introduced AMH as one of the factors involved in the inhibition of primary follicle growth and its response to FSH [27–29]. Although the exact pathophysiology of AMH in human ovarian aging is still a matter of debate, it is well documented that AMH is an appropriate biomarker of ovarian reserve and function; as a result lower AMH may be associated with an increase in vascular risks and events [4, 30–36]. Koninger et al. showed that AMH levels decrease during pregnancy [37]; in 2015; they also confirmed that this decline is real and that it is not due to interfering complement activation with the conventional assa**y** [38].

Yarde et al. demonstrated a lower AMH level in women with a history of preeclampsia, suggesting that both PE and premature ovarian aging are manifestations of underlying vascular dysfunction [14]; however, the researchers in this study used AMH, not age-specific AMH, in their analysis; previous investigators preferred using age-specific AMH, rather than AMH, as a better predictor of ovarian reserve status [39]. Similarly, Shand et al., in a retrospective study found that women with a very low AMH (1.5 pmol/L) in early pregnancy may have a slightly increased risk for subsequent PE [15]. However, interpretation of these data is difficult, since women with significantly lower levels of AMH (and thus potentially at highest risk for subsequent pregnancy-related hypertensive disease) would also be less likely to conceive, thus potentially being excluded from the population on their bases.

Finally, a prospective study by Tokmak et al. in 2015 reported maternal serum AMH to be lower in PE patients than in those with normal pregnancies, albeit with low sensitivity and specificity (AUC = 0.59, sensitivity = 67.4 and specificity = 47.1%) for the prediction of PE [16]; they also used the absolute maternal AMH, rather than the age-specific AMH.

In contrast, Birdir, et al. investigated the AMH levels at 11–13 weeks of gestation in prediction of PE and concluded that maternal serum AMH is not an effective predictor of PE in pregnancy [40]. Since AMH levels during pregnancy are often variable [37, 40], Since AMH levels during pregnancy are often variable [35, 38], which could influence relationship between AMH and PE, we assessed this association in non-pregnant women. However, similar to Birdir study findings, the current study did not show any association between AMH and PE. We concluded that while a sizeable body of evidence exists regarding the relationship of AMH and the development of PE, most studies using age-specific AMH levels have shown no association, or a weak association.

Our study has number of strengths. It is unique in that it uses unselected pregnant women in a subset of a cohort study with a long-term follow-up. Also, in our analyses we used ‘age-specific’ AMH, and our method for identification of age-specific AMH was precise. As we have shown, among all available models, our model (exponential–normal (EN) three-parameter model) provided a good fit, since the normal plot of the Z-scores from our model appears reasonably linear; moreover, in our model about 10% of the observations lie above the 90th percentile or below the 10th percentile. Another strength of our study was that laboratory measurements were done simultaneously at the same laboratory by the same person in order to minimize intra-assay variability in the data. Since all blood samples were centrifuged within 30–45 min of collection and stored at − 80 degrees C, AMH molecular instability was not an issue in this study [34].

There were also some limitations to our study; one potential limitation was that we had only one measurement of AMH per case in our cohort. Another limitation was that due to design of the study we were unable to discuss causality and effect. We acknowledge that recall bias in self-reporting of preeclampsia might be another limitation; however, we see no reason to believe that there would be a differential recall bias among women based on their AMH levels; a similar rate of recall error would be expected in both groups and this would not impact our results. Moreover, there is a body of literature that suggests the reliability of self-reporting PE as a variable in epidemiologic studies using standard questionnaires [18, 19]. The time interval between AMH measurement and occurrence of pregnancy or PE was not collected in details, as a result we are unable to adjust our results for this time interval; despite that a single AMH measurement can precisely identify the ovarian reserve status of subjects as its level remains almost constant from one cycle to another and has a high intraclass correlation coefficient as a result of which only one measurement provides a reliable estimate of its mean in each woman [41]. Finally, since we had not measured pre-conception AMH, given the potential decline in fertility rates of women with lower AMH levels and any possible changes in these levels overtime, our values were not entirely reflective of levels preceding the index pregnancy and need to be interpreted cautiously.

## Conclusions

Age-specific AMH may not be a suitable marker for prediction of PE. Further longitudinal studies, considering pre-conception measurement of AMH, are recommended for better interpretation of the association between ovarian reserve status and PE.

## Data Availability

The dataset used during the current study is available from the corresponding author on reasonable request.

## Abbreviations

AMH: Anti-Mullerian Hormone
apo B: Apolipoprotein B
AUC: Area under curve
BMI: Body mass index
CVs: COEFFICIENTS of Variations
DBP: Diastolic Blood Pressure
EIA: Enzyme immunoassay
EN: Exponential–normal
FPG: Fasting plasma glucose
HDL-C: High-density Lipoprotein Cholesterol
LDL-C: Low-density Lipoprotein Cholesterol
PE: Pre-Eclampsia
SBP: Systolic Blood Pressure
TC: Total Cholesterol
TG: Triglyceride
TLGS: Tehran Lipid and Glucose Study
WC: Waist Circumference
